# 2023 Korean Society of Echocardiography position paper for diagnosis and management of valvular heart disease, part I: aortic valve disease

**DOI:** 10.1186/s44348-024-00019-0

**Published:** 2024-07-26

**Authors:** Sun Hwa Lee, Se-Jung Yoon, Byung Joo Sun, Hyue Mee Kim, Hyung Yoon Kim, Sahmin Lee, Chi Young Shim, Eun Kyoung Kim, Dong-Hyuk Cho, Jun-Bean Park, Jeong-Sook Seo, Jung-Woo Son, In-Cheol Kim, Sang-Hyun Lee, Ran Heo, Hyun-Jung Lee, Jae-Hyeong Park, Jong-Min Song, Sang-Chol Lee, Hyungseop Kim, Duk-Hyun Kang, Jong-Won Ha, Kye Hun Kim, on the behalf of the Korean Valve Survey Investigators, Sun Hwa Lee, Se-Jung Yoon, Byung Joo Sun, Sahmin Lee, Chi Young Shim, Eun Kyoung Kim, Jun-Bean Park, Jeong-Sook Seo, Jung-Woo Son, In-Cheol Kim, Hyungseop Kim, Kye Hun Kim, Wook-Jin Chung, Hyo-Suk Ahn, Woo-Baek Chung, Eun Joo Cho, Jung Sun Cho, Dong Ryeol Ryu, Dong Heon Yang, Jeong Rang Park, Woo-Shik Kim, Il Suk Sohn, Jin Oh Na, Seong-Mi Park, Sun Ho Hwang, Ji-Yong Choi, Tae-Ho Park, Yong Hyun Park, Jung Hyun Choi, Hack-Lyoung Kim, Jun-Bean Park, Jin-Sun Park, Eui-Young Choi, Jang-Won Son, Shin-Jae Kim, Sang Jae Rhee, In-Jeong Cho, Young Sup Byun, Jeong-Sook Seo, Sung-Hee Shin, Jong Wook Beom, Ju-Hee Lee, Dae-Hwan Bae, Sung-Ai Kim, Dae Gyun Park, Min-Kyung Kang, Kyung-Soon Hong, Ran Heo

**Affiliations:** 1 Division of Cardiology, Department of Internal Medicine, Jeonbuk National University Medical School, Jeonbuk National University Hospital, Jeonju, Republic of Korea; 2 Division of Cardiology, National Health Insurance Service Ilsan Hospital, Goyang, Republic of Korea; 3 Division of Cardiology, Asan Medical Center, University of Ulsan College of Medicine, Seoul, Republic of Korea; 4 Division of Cardiology, Department of Internal Medicine, Chung-Ang University Hospital, Chung-Ang University College of Medicine, Seoul, Republic of Korea; 5 Department of Cardiovascular Medicine, Chonnam National University Hospital, Chonnam National University Medical School, Gwangju, Republic of Korea; 6 Division of Cardiology, Severance Cardiovascular Hospital, Yonsei University College of Medicine, Seoul, Republic of Korea; 7 Division of Cardiology, Department of Medicine, Samsung Medical Center, Sungkyunkwan University School of Medicine, Seoul, Republic of Korea; 8 Heart Vascular Stroke Institute, Samsung Medical Center, Seoul, Republic of Korea; 9 Division of Cardiology, Department of Internal Medicine, Korea University Anam Hospital, Seoul, Republic of Korea; 10 Division of Cardiology, Department of Internal Medicine, Seoul National University Hospital, Seoul, Republic of Korea; 11 Division of Cardiology, Department of Internal Medicine, Inje University Busan Paik Hospital, Inje University College of Medicine, Busan, Republic of Korea; 12 Division of Cardiology, Department of Internal Medicine, Yonsei University Wonju College of Medicine, Wonju, Republic of Korea; 13 Department of Internal Medicine, Keimyung University Dongsan Medical Center, Daegu, Republic of Korea; 14 Division of Cardiology, Pusan National Yangsan Hospital, Pusan National University School of Medicine, Busan, Republic of Korea; 15 Research Institute for Convergence of Biomedical Science and Technology, Pusan National Yangsan Hospital, Busan, Republic of Korea; 16 Division of Cardiology, Department of Internal Medicine, Hanyang University College of Medicine, Seoul, Republic of Korea; 17 Division of Cardiology, Department of Internal Medicine, Chungnam National University Hospital, Chungnam National University College of Medicine, Daejeon, Republic of Korea; 18 Regional Cardiocerebrovascular Center, Chungnam National University Hospital, Daejeon, Republic of Korea

**Keywords:** Aortic stenosis, Aortic regurgitation, Echocardiography

## Abstract

This manuscript represents the official position of the Korean Society of Echocardiography on valvular heart diseases. This position paper focuses on the clinical management of valvular heart diseases with reference to the guidelines recently published by the American College of Cardiology/American Heart Association and the European Society of Cardiology. The committee tried to reflect the recently published results on the topic of valvular heart diseases and Korean data by a systematic literature search based on validity and relevance. In part I of this article, we will review and discuss the current position of aortic valve disease in Korea.

## Background

The purpose of this position paper is to support healthcare professionals in selecting the best management strategies for individual patients by assessing each patient’s clinical condition, considering the risks and benefits of a specific diagnosis and treatment methods, and understanding the likely effects on the outcome. Many academic societies and organizations have published guidelines and recommendations for various diseases to facilitate the decision-making of healthcare professionals. Because established guidelines significantly influence clinical decisions, the quality standards for the established guidelines should be defined so that all decision-making processes can be transparently disclosed.

This position paper represents the official position of the Korean Society of Echocardiography (KSE) on valvular heart diseases and will be revised regularly. The recommendations were prepared to help healthcare professionals make decisions in their daily practice by evaluating and summarizing the existing evidence about clinical problems, but the final decision for each individual patient should be made by healthcare providers after sufficient discussion and negotiation with the patient and their caregivers.

To develop this position paper, professional members of the guideline-making committee of the KSE, all experts in the field of valvular heart disease, were selected. The selected experts comprehensively reviewed published evidence associated with the management of valvular heart disease (diagnosis, treatment, prevention, and rehabilitation, etc.). This position paper focuses on the clinical management of valvular heart diseases by referring to guidelines recently published by the American College of Cardiology/American Heart Association (ACC/AHA) [1] and the European Society of Cardiology/European Association of Cardio-Thoracic Surgeons (ESC/EACTS) [2]. The committee conducted a systematic literature search based on validity and relevance to ascertain the recently published results on the topic of valvular heart diseases. This position paper was developed after careful consideration of recently available scientific and clinical knowledge and evidence. The writing committee reviewed this paper carefully and revised it to ensure overall uniformity. Then, it was reviewed by external experts from other related scientific societies and revised based on their suggestions. The final documents will be published on the KSE website. The committee members who participated in drafting and reviewing this position paper disclosed all interests that could be potential conflicts. This position paper was created without financial support from related industries to exclude their interests.

In the future, follow-up investigations will be conducted to verify whether daily clinical practice follows the recommendations in this position paper. It is necessary to establish a cycle of clinical research, preparation and dissemination of guidelines, clinical application, and revision of guidelines to reflect clinical practice. Although this position paper should be applied in daily clinical practice to prevent, diagnose, and treat valvular heart diseases, individual clinicians have both the right and the responsibility to make clinical decisions in consultation with patients and guardians and with full consideration of each patient’s clinical condition. In addition, it is the responsibility of individual healthcare providers to check related regulations, such as the need for various approvals and insurance benefits, that might influence choices about medications and devices. In these two articles, we review and share current information about valvular heart disease in Korea; part I focuses on aortic stenosis and regurgitation, and part II will focus on mitral regurgitation and stenosis and tricuspid regurgitation.

## Aortic stenosis

### Etiology

Aortic stenosis (AS) is the most common valvular disease leading to surgical or transcatheter valve replacement [3]. The prevalence of AS is increasing due to the aging population [4, 5]. In addition to its degenerative etiology, which is increasing rapidly in developed countries, the rheumatic etiology is still frequently observed in many parts of the world [3, 4]. Bicuspid aortic valve (BAV), a common congenital valve anomaly affecting 0.5% to 2.0% of adults with a 3:1 male to female predominance, is common in younger patients with AS [6].

### Stages

Disease progression is classified based on patient symptoms, valve anatomy, hemodynamic severity, and left ventricular (LV) and vascular response. Table 1 shows the stages of AS, ranging from patients at risk of AS (stage A) or with progressive hemodynamic obstruction (stage B) to severe asymptomatic (stage C) and symptomatic AS (stage D) [1]. The severity of valve dysfunction is best characterized by the maximum transaortic velocity or mean pressure gradient at the normal transaortic flow rate. Some patients with AS have a low transaortic flow rate because of LV systolic dysfunction with a low LV ejection fraction (EF) or because of a small, hypertrophied LV with low stroke volume (SV). Severe AS with low flow is classed as D2 (with low LVEF) or D3 (with normal LVEF). Stage D4 AS is defined as normal-flow, low-gradient, symptomatic AS with preserved LVEF. Careful attention to detail is required when assessing valvular hemodynamics, either by Doppler echocardiography or cardiac catheterization, and the inherent variability of measurements and calculations should always be considered in clinical decision-making.

**Table 1:** Stages of aortic stenosis

Characteristic	Stage							
	A	B		C1	C2	D1	D2	D3
Definition	At risk	Progressive		Asymptomatic severe		Symptomatic severe		
Severity	Normal to trivial	Mild	Moderate	Severe				
Echocardiography								
Morphology								
Leaflet	BAV, sclerosis	Calcified, fibro-thickening, commissure fusion		Severe calcified, fibro-thickening, commissure fusion				
Motion	Normal	Mild to moderate systolic motion reduction		Severely reduced opening				
AVA-CE (cm^2^)	-	1.5–2.0	1.0–1.5	≤1.0	≤1.0	≤1.0	≤1.0^a^	≤1.0
Vmax (m/sec)	<2.0	2.0–2.9	3.0–3.9	≥4.0	≥4.0	≥4.0	<4.0^a^	<4.0
MSPG (mmHg)	-	<20	20–39	≥40	≥40	≥40	<40	<40
AVAi (cm^2^)	-	≥1.0	0.6–0.9	<0.6				
LV diastolic dysfunction	None	Early		Mild		Significant		Restrictive
LV hypertrophy	None	Mild		Mild		Significant		↑RWT, ↓Cavity
LVEF	Normal	Normal		Normal	<50	Normal	<50	Normal^b^
Flow-gradient	Normal	Normal		NF-HG	NF-HG	NF-HG	LF-LG	NF-LG/pLF-LG
Symptom	None	None		None		DOE, EI, cardinal symptoms^c^		

*BAV* Bicuspid aortic valve, *AVA-CE* Aortic valve area by continuity equation, *Vmax* Peak aortic jet velocity, *MSPG* Mean systolic pressure gradient, *AVAi* Aortic valve area index, *LV* Left ventricular, *RWT* Relative wall thickness, *LVEF* Left ventricular ejection fraction, *NF* Normal flow, *HG* High gradient, *LF* Low flow,* LG* Low gradient, *pLF* paradoxical low flow, *DOE* Dyspnea on exertion, *EI* Exercise intolerance

^a^AVA < 1.0 cm^2^ with AV Vmax > 4.0 m/sec during dobutamine stress echocardiography in D2

^b^Stroke volume index < 35 mL/m^2^

^c^Cardinal symptoms include heart failure, angina, presyncope, and syncope

### Korean data

Among patients with valvular heart disease (VHD) in the nationwide retrospective cohort for the Korean Valve Survey [7], nearly 30% had moderate to severe AS (mean age, 76 ± 11 years; 47% male sex), and the most common comorbidity in patients with AS was hypertension (67%). LV size (LV end-diastolic dimension [LVEDD], 49 ± 7 mm; LV end-systolic dimension [LVESD], 32 ± 8 mm) and systolic function (LVEF, 60% ± 12%) were generally within the normal range, and only 9% had an LVEF of ≤ 40%. As measures of AS severity, peak transaortic velocity and mean transaortic pressure gradient were 3.9 ± 0.9 m/sec and 37 ± 19 mmHg, respectively, and aortic valve area was 1.0 ± 0.2 cm^2^ by two-dimensional (2D) planimetry and 1.0 ± 0.3 cm^2^ by the continuity equation method. Degenerative disease was the most common cause of AS (79%), followed by congenital disease (e.g., BAV; 9%) and rheumatic disease (8%).

Important studies on early intervention in asymptomatic patients with severe AS have been conducted in Korea and have influenced the recent guidelines [1, 2]. In an observational study, Kang et al. [8] demonstrated the benefit of early surgery in asymptomatic patients with very severe AS, defined as critical stenosis in aortic valve area (≤ 0.75 cm^2^) accompanied by peak aortic jet velocity ≥ 4.5 m/sec or mean transaortic pressure gradient ≥ 50 mmHg. They showed that early surgery was associated with significantly lower 6-year cardiac mortality (0% in the surgical group vs. 24% ± 5% in the conventional treatment group, *P* < 0.001) and all-cause mortality (2% ± 1% in the surgical group vs. 32 ± 6% in the conventional treatment group, *P* < 0.001). In 57 propensity score-matched pairs, the risk of all-cause mortality was significantly lower in the surgical group than in the conventional treatment group (hazard ratio [HR], 0.135; 95% confidence interval [CI], 0.030–0.597; *P* = 0.008).

Recent results from a prospective randomized controlled trial (RCT) comparing early surgery with conservative management also confirm the benefit of early surgery in asymptomatic patients with very severe AS. In the RECOVERY trial, Kang et al. [9] showed that the incidence of the composite outcome of operative mortality or death from cardiovascular causes during follow-up was significantly lower in those who underwent early aortic valve replacement (AVR) surgery than in those who received conservative care. The primary endpoint event occurred in one patient in the early surgery group (1%) and in 11 of 72 patients (15%) in the conservative care group (HR, 0.09; 95% CI, 0.01–0.67; *P* = 0.003). Death from any cause occurred in five patients (7%) in the early surgery group and 15 patients (21%) in the conservative care group (HR, 0.33; 95% CI, 0.12–0.90). In the conservative care group, the cumulative incidence of sudden death was 4% at 4 years and 14% at 8 years.

### Diagnosis and follow-up

#### Echocardiography

Transthoracic echocardiography (TTE) is the standard diagnostic tool for AS. A comprehensive TTE examination for AS should include not only stenosis severity, but also LV function, LV wall thickness, size of the left atrium, diameter of the LV outflow tract (LVOT), any abnormal structure in the LVOT, and anatomy of the aortic root [10, 11]. In AS, the aortic valve (AV) typically shows thickening, stiffening, and calcification, with some different features according to underlying etiology. The most common etiology, degenerative AS, usually presents with prominent calcification in the middle of the cusp tips. BAV-related AS presents with two asymmetrical cusps with an ovoid valvular orifice [10, 12]. Rheumatic AS shows commissural fusion and is usually combined with mitral valve pathologies [10, 13]. In BAV-related AS and rheumatic AS, the AV frequently shows systolic doming; thus, the AV opening in short-axis images can be overestimated compared with the true orifice, and examiner caution is required.

The key echocardiographic parameters of AS are peak velocity, mean pressure gradient (MG), and AV area (AVA) [10]. For hemodynamic assessment of AS, normal SV is an important condition [14, 15]. If hypertension is combined with AS, it is an additional afterload on the LV, which can reduce both the SV and the pressure gradient across the AV [16, 17]. Therefore, hypertension should be controlled before echocardiographic evaluation for AS [1]. To assess peak velocity, the Doppler beam should be parallelized with blood flow across the AV [10]. The peak velocity should be measured through every available location, such as the LV apical, right parasternal, suprasternal, and subcostal windows [10].

Patients who are diagnosed with severe AS (defined as AVA ≤ 1 cm^2^) are subdivided into four hemodynamic categories according to MG, SV indexed by body surface area (SVi), and LVEF: (1) high-gradient AS (MG ≥ 40 mmHg, peak velocity ≥ 4.0 m/sec); (2) low-flow, low-gradient AS with reduced EF (MG < 40 mmHg, LVEF < 50%, SVi ≤ 35 mL/m^2^); (3) low-flow, low-gradient AS with preserved EF (MG < 40 mmHg, LVEF ≥ 50%, SVi ≤ 35 mL/m^2^); and (4) normal-flow, low-gradient AS with preserved EF (MG < 40 mmHg, LVEF ≥ 50%, SVi > 35 mL/m^2^) (Table 1) [2, 18]. In high-gradient AS, the diagnosis is clear because the reduced AVA and increased MG are concordant. On the other hand, low-gradient AS is a condition in which MG is underestimated due to the reduced flow rate despite reduced AVA. It is defined as “classical low flow” if the origin of the low flow is reduced LVEF (< 50%) [2]. In this situation, low-dose dobutamine stress echocardiography can be considered for definite diagnosis [1, 2, 19]. If AV peak velocity and MG are both increased (≥ 4 m/sec and ≥ 40 mmHg, respectively) with increased flow rate caused by dobutamine stress and the reduced AVA is fixed (≤ 1 cm^2^), severe AS can be diagnosed [1, 2]. In contrast, if the AVA increases with increased flow rate but MG remains lower than severe AS, it can be diagnosed as pseudo-severe AS [1, 2]. If the increase in SV does not reach 20% of baseline, it is determined to be “lack of contractile (or flow) reserve” [20, 21]. In low-gradient AS with preserved EF, measurement error should first be excluded because it is important to avoid underestimating the LVOT diameter [10]. After measurement error is excluded, common clinical factors that cause low flow status are old age, small body size, high blood pressure, severe LV hypertrophy, and diastolic dysfunction [14, 15, 21, 22]. Significant mitral regurgitation, tricuspid regurgitation, right ventricular dysfunction, and ventricular septal defects are also causal factors for reduced SV [2, 23, 24]. Normal-flow, low-gradient AS with preserved EF shows a clinical course similar to that of moderate AS [2, 25]. As such, confirming severe AS can be difficult in situations with discordant hemodynamic parameters. The most important points are the physician’s assessment of the patient’s abnormal symptoms and whether they are relevant to the severity of AS [1, 2, 12]. Other echocardiographic parameters, the Doppler velocity index (dimensionless index) [26], and LV global longitudinal strain [27, 28] can also be used in diagnosis. An elevated serum B-type natriuretic peptide level more than threefold of the age- and sex-corrected normal range is also an important clue for significant LV loading [29, 30]. In cases that remain unclear despite conventional testing, multimodality imaging should be considered.

#### Multimodality imaging

In asymptomatic patients with AS, exercise testing is a useful tool for identifying hidden symptoms [1, 2, 31]. Patient symptoms are subjective, and AS detection in elderly patients is especially difficult due to their low level of physical activity. In this situation, exercise testing is useful and can be applied directly to clinical decision-making: AVR is recommended for patients with severe AS who develop clear symptoms or hemodynamic abnormalities during exercise testing [1, 2]. Safety issues with exercise testing in AS have been addressed, and it can be performed safely under supervision by an experienced physician [31, 32]. For patients with symptomatic AS, on the other hand, exercise testing is contraindicated because frequent adverse events have been reported [1, 33].

In cases in which TTE returns discrepant hemodynamic parameters, other imaging modalities need to be considered. Transesophageal echocardiography (TEE) is frequently used for 2D measurement of the AVA (direct planimetry) [10]. This is a very useful approach in cases with a heavily calcified AV, in which planimetric assessment by TTE can be limited. Additionally, TEE can be used for the combined assessment of aortic regurgitation, mitral valve function, and anatomy of the aortic root [10]. Cardiac computed tomography (CT) is a unique modality for quantifying vascular and valvular calcification [34, 35]. The AV calcification score is an additional diagnostic parameter for severe AS, and it is a powerful predictor of adverse clinical outcomes [1, 2, 34, 35]. Diagnostic criteria for severe AS are defined based on sex. The cutoff points for Agatston units in men and women are 2,000 and 1,300, respectively [1]. Additionally, the accurate aortic diameter, which is perpendicular to the long axis of the aorta, can be measured from 3D CT images and is useful for detecting combined aortic aneurysms [36]. Cardiac magnetic resonance imaging (CMR) is a unique modality for quantifying ventricular fibrosis, and its parameters have been reported to be associated with the long-term clinical outcomes of AS [37–40]. As an invasive diagnostic modality, cardiac catheterization is not frequently used for purely diagnostic purposes. It is considered for cases in which the diagnosis from non-invasive imaging modalities remains inconclusive [20, 41]. It has an additional role of assessing coronary anatomy and can be performed when planning AVR [1].

To prepare for transcatheter AV replacement (TAVR), special imaging studies are required [2]. Cardiac CT is a representative imaging modality that can comprehensively assess the AV structure, severity of calcification, LVOT diameter, appropriate size for the prosthetic valve, distance between the annuls plane and coronary ostium, and compatibility of vascular access [2]. TEE is a very useful modality for real-time monitoring of the procedure because it can demonstrate the hemodynamics and occurrence of paravalvular leakage after valve implantation [42].

#### Follow-up

The progression of AS varies according to the patient’s clinical factors, but rapid deterioration is common after development of symptoms [12]. Therefore, it is important to instruct patients to promptly visit the clinic at symptom initiation [2]. Follow-up echocardiography for AS is needed periodically because a patient’s symptoms are frequently subjective and have ambiguous progression [1, 2]. It is recommended that follow-up TTE should be performed every 6 months (at least) in patients with severe AS, every 12 months in patients with moderate AS, and every 2 to 3 years in those with mild AS [2]. Moreover, in situations of hemodynamic fluctuation (e.g., major surgery, pregnancy, systemic infection, significant bleeding, anemia), additional TTE is reasonable to optimize loading conditions and systemic circulation [1]. AV sclerosis is a condition of cusp thickening and calcification without meaningful stenosis (AV peak velocity < 2.0 m/sec), but its progression rate to significant AS is not negligible (10% in 5 years) [1, 43]. Thus, it is reasonable to consider patients with AV sclerosis as targets for clinical follow-up [1].

### Medical therapy

Currently, no medical therapy for AS improves its clinical prognosis or modifies its progression [1, 2, 12]. For patients with AS and coexisting hypertension, control of blood pressure according to hypertension guidelines is recommended to prevent additional hemodynamic load on the LV [1, 2, 44, 45]. No evidence supports the use of a specific antihypertensive medication for AS. However, diuretics are not recommended due to concerns about reduced SV and excessive reduction of blood pressure [1].

A large RCT, Simvastatin and Ezetimibe in Aortic Stenosis, reported that statins could not delay the progression of AS [46]. However, the patient group under statin therapy showed a significantly lower ischemic event rate; thus, statin therapy in AS is reasonable for primary and secondary prevention of concomitant coronary artery disease [1, 46–49]. Among patients undergoing TAVR, those taking renin-angiotensin system inhibitors showed significantly lower frequencies of mortality and heart failure than the control group [50, 51]. An RCT tested anticalcifying agents for AS and found that denosumab and bisphosphonate showed no significant effects in delaying AS progression compared with placebo [52]. However, in a recent Korean retrospective study, the use of dipeptidyl peptidase-4 inhibitors with favorable heart to plasma concentration ratios and anticalcification ability reduced the risk of AS progression [53].

### Timing of intervention

**Fig. 1:**
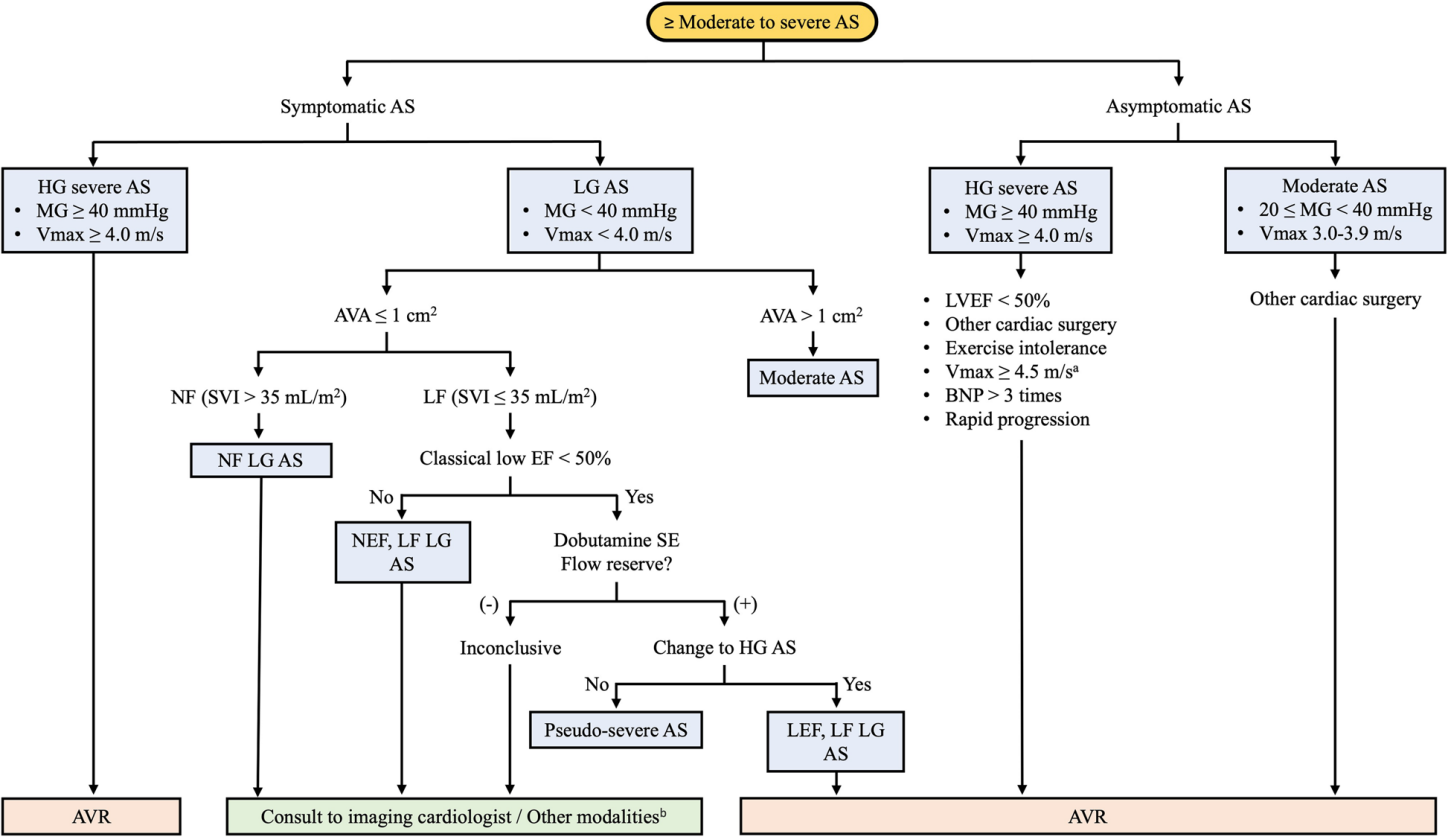
Treatment of aortic stenosis (AS): timing of intervention. AVA, aortic valve area; AVR, aortic valve replacement; BNP, B-type natriuretic peptide; EF, ejection fraction; HG, high gradient; LEF, low ejection fraction; LF, low flow; LG, low gradient; LVEF, left ventricular ejection fraction; MG, mean pressure gradient; NEF, normal ejection fraction; NF, normal flow; SE, stress echocardiography; SVI, stroke volume index; Vmax, peak aortic jet velocity. ^a^The American College of Cardiology/American Heart Association guideline [1] and the European Society of Cardiology guidelines [2] recommend AVR for asymptomatic AS patients with Vmax ≥ 5.0 m/sec. ^b^Transesophageal echocardiography, cardiac computed tomography with aortic valve calcium score, or cardiac magnetic resonance imaging should be considered

In the current guidelines, symptoms related to VHD should be present before considering surgical AV replacement (SAVR) or TAVR in patients with severe AS. The schematic in Fig. 1 shows that symptoms are the first criterion when considering the need for intervention [1, 2].

Nonetheless, the guidelines recommend intervention for very severe AS (MG ≥ 60 mmHg or peak aortic jet velocity [Vmax] > 5 m/sec) with LVEF > 55% and a normal exercise test even in the absence of symptoms [1, 2]. Kang et al. [8, 9] reported the results of a trial involving patients with asymptomatic, very severe AS, defined as Vmax ≥ 4.5 m/sec or MG ≥ 50 mmHg, randomized to SAVR or conservative management (clinical follow-up and observation). The outcomes were significantly better for patients who underwent SAVR promptly (within approximately 2 months of randomization) than for those randomized to conservative care.

A meta-analysis of early intervention versus conservative management for severe, asymptomatic AS supports the association of early intervention for patients with severe, asymptomatic AS with reduced all-cause, cardiovascular, and noncardiovascular mortality during follow-up without an increase in any procedure-related clinical outcomes [54]. A large body of data indicates that many patients with severe, asymptomatic AS develop an indication for AV intervention, and their deaths are mostly of cardiac origin, including sudden cardiac arrest [2, 55–58].

A study using CMR to guide early AVR in patients with severe, asymptomatic AS is also being conducted. The EVOLVED study is the first multicenter RCT to compare early AVR guided by the presence of a focal scar on late gadolinium enhancement imaging to routine care in severe, asymptomatic AS [59]. If this study demonstrates a role for CMR in guiding the timing of intervention, then the next target for CMR-guided early intervention will be moderate AS with evidence of myocardial decompensation [60]. Everett et al. [61] asserted that it is ideal to intervene in patients with severe AS just as the LV is starting to decompensate but before substantial irreversible damage has accrued, which they judged to be the time at which the short-term and long-term risks of intervention are outweighed by the risks of not intervening.

However, arguing for caution about early AVR, Lancellotti and Vannan [62] suggested that instead of directly extending existing results to patients with severe, asymptomatic AS, we should wait for guidance from the results of large, randomized studies of early TAVR in patients with severe, asymptomatic AS. In the real clinical field, optimizing the timing of AV intervention for severe AS is very difficult. Management of severe, asymptomatic AS is controversial, and the decision to intervene requires careful assessment of the benefits and risks for each patient [2].

We also take much interest in moderate AS. A recent study presented results of early operations for moderate AS. Among patients with moderate AS, those with decreased LVEF or SVi are at high risk. Patients with elevated E/e’ ratios are at intermediate risk even if the two parameters are preserved. The authors of that study recommend further investigation to assess whether earlier intervention could improve outcomes and reduce cardiac-related death among patients at high and intermediate risk [63]. A study by Jean et al. [64] analyzed patients with both heart failure with reduced EF (HFrEF) and moderate AS. That multicenter retrospective study included 262 patients diagnosed with both HFrEF (defined as LVEF < 50%) and moderate AS and reported that the conditions were associated with a marked incremental risk of mortality. AVR (especially TAVR) during follow-up was associated with improved survival in patients with HFrEF and moderate AS [64, 65]. In patients with moderate AS, follow-up and echocardiographic evaluation for symptoms and EF are very important.

### Choice of intervention

The current guidelines from the ACC/AHA [1] and ESC/EACTS [2] recommend AV intervention for patients diagnosed with severe symptomatic or asymptomatic AS [1, 2].

The selection of a prosthetic valve type is influenced by several factors, including patient age, values, and preferences; expected bioprosthetic valve durability, avoidance of patient–prosthesis mismatch, the potential need for and timing of reintervention; and the risks associated with long-term vitamin K antagonist anticoagulation therapy with mechanical valve replacement [1]. Despite the significantly higher rate of structural valve deterioration observed with bioprostheses in younger patients compared with older patients [56, 57, 60, 62, 66–70], many patients choose to avoid mechanical prostheses because they do not want to undergo long-term vitamin K antagonist therapy, which involves the inconvenience of monitoring, dietary restrictions, medication interactions, and the need to restrict participation in some types of athletic activity. A mechanical valve might be a prudent choice for patients for whom a second surgical procedure would involve very high risk (e.g., those with porcelain aorta or prior radiation exposure). The availability of TAVR has changed the choice between mechanical and bioprosthetic valves for younger patients [71–77].

**Fig. 2:**
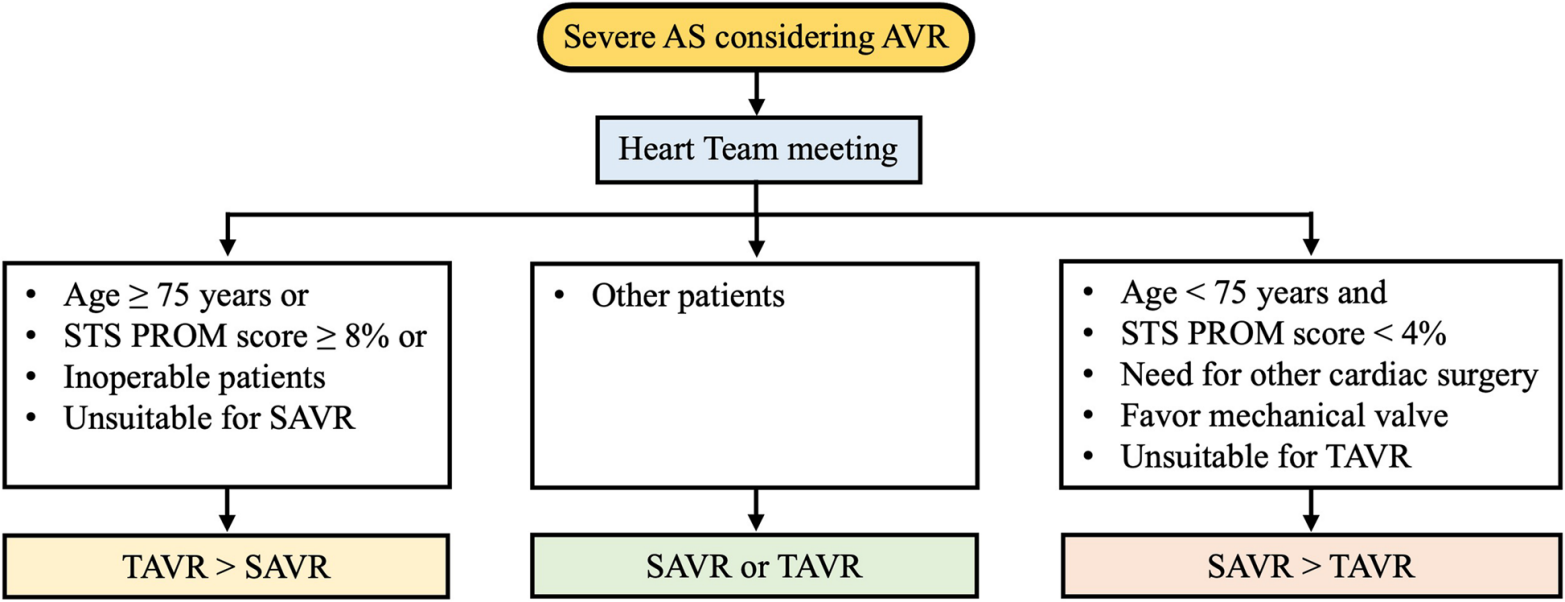
Treatment of aortic stenosis (AS): choice of intervention. AVR, aortic valve replacement; SAVR; surgical aortic valve replacement; STS, Society of Thoracic Surgeons; PROM, 30-day predicted risk of mortality score; TAVR, transcatheter aortic valve replacement

In patients considering a bioprosthetic, the next step is the choice between SAVR and TAVR (Fig. 2). In patients whose risk for SAVR is high or prohibitive, decision-making focuses on TAVR versus palliative care. When the surgical risk is not high or prohibitive, the merits and demerits of each procedure are assessed. When both SAVR and TAVR are options, a prime consideration is the limited data about TAVR durability. SAVR has been used for more than 50 years, and ample durability data are available for specific valve types across age groups. Currently, robust durability data for TAVR extend to only about 5 years. SAVR valve deterioration typically occurs after > 10 years, so longer-term TAVR durability data are needed. A key factor in decision-making is the ratio of patient life expectancy to known valve durability, with patient age often used as a surrogate for life expectancy [1, 2]. Many large multicenter, randomized clinical trials comparing TAVR with standard therapy and SAVR have been performed in high-risk, intermediate, and low-risk patients with severe AS [74–81]. Although it has not been tracked for a long time, TAVR has shown high clinical value and safety.

Decision-making should be individualized based on patient-specific factors that affect longevity or quality of life, such as comorbid cardiac and noncardiac conditions, frailty, and dementia. The implantation approach should be chosen in a shared decision-making process that considers patient values and preferences and includes a discussion of the indications for and against each approach and the potential need for and risks associated with valve reintervention [1, 2, 82–85].

In summary, prosthetic heart valve durability is a key consideration in younger patients (< 75 years) at low surgical risk, and SAVR (if feasible) is the preferred treatment option. Conversely, durability is a lower priority in older patients (≥ 75 years) and those who are inoperable or at high risk for surgery, and TAVR is preferred in those groups (particularly if it is feasible via the transfemoral approach). The Heart Team should make tailored recommendations for remaining patients based on their individual characteristics. This guidance should be readdressed when further data about the long-term durability of TAVR become available [1].

Although several registries have reported excellent outcomes from TAVR in patients with a BAV who were unsuitable for surgery [86, 87], SAVR remains more appropriate in patients with AS affecting a BAV and in those with associated disease (e.g., aortic root dilatation, complex coronary disease, or severe mitral regurgitation) requiring surgery [1]. Many studies on the incidence and characteristics of VHD, including BAV, have been performed in Korea [88–92]. In addition, TAVR procedures are performed in many centers in Korea, and many studies on TAVR have been conducted [93–97].

## Aortic regurgitation

### Etiology

Aortic regurgitation (AR) can have acquired and congenital causes and results in failure of the cusps to coapt. This condition can induce symptoms of heart failure, and the regurgitant volume causes direct overload on the LV, ultimately leading to LV decompensation.

The etiology of AR can be categorized into primary AV diseases and secondary aortic root abnormalities. Primary AV diseases causing AR include degenerative calcific, rheumatic, and congenital valvular anomalies [98, 99], with BAV being the most common congenital AV anomaly. Unicuspid or quadricuspid valves are rare causes of AR. Acute AR can be related to infective endocarditis or blunt chest trauma, and iatrogenic AV injury during transcatheter procedures is emerging as a cause of acute AR in high-income countries [100]. Aortic root diseases involving the aortic sinuses or ascending aorta, including those in connective tissue diseases such as Marfan syndrome or Ehlers-Danlos syndrome, sinus of Valsalva aneurysm, or proximal aortitis related to Behçet disease, Takayasu arteritis, or ankylosing spondylitis, can also be associated with AR. In aortic dissection, acute dilatation of the aortic root, often combined with prolapse of the dissection flap, results in acute AR.

Degenerative calcific valves, BAV, and aortic root abnormalities are common etiologies of chronic AR in high-income countries, whereas rheumatic AR is the most common cause of AR in developing countries [99]. Chronic AR typically develops in patients with abnormalities of AV anatomy or aortic root geometry and progresses slowly. In patients with moderate to severe chronic AR, the LV gradually dilates due to volume overload, which is eventually followed by impairment of LV systolic function, particularly in those with severe AR [99].

### Stages

The stages of chronic AR are based on valve anatomy and hemodynamics, LV size and function, and symptoms (Table 2) [1]. Stage A of AR includes patients at risk of AR but with no or trace AR and no symptoms. Stage B is mild to moderate AR with compensated LV and no symptoms. Stage C indicates severe, asymptomatic AR with compensated LV (C1) or decompensated LV (C2). Severe, symptomatic AR is called stage D, regardless of LV compensation status. The severity of AR is assessed based on jet width, vena contracta, regurgitant volume, regurgitant fraction, effective regurgitant orifice, and angiographic grade.

**Table 2:** Stages of chronic aortic regurgitation

Characteristic	Stage					
	A	B		C1	C2	D
Definition	At risk	Progressive		Asymptomatic severe		Symptomatic severe
Severity	Normal to none	Mild	Moderate	Severe		
Echocardiography						
Morphology						
Leaflet	Congenital, BAV, sclerosis	Calcification or rheumatic changes		Severe calcification or rheumatic changes		
Sinus/ascending aorta	-	Dilated		Dilated		
Vena contracta width (mm)	-	<3	3–6	≥6		
Jet width (% of LVOT)	-	<25	25–64	≥65		
JCSA (% of LVOT)	-	<5	5–59	≥60		
EROA (cm^2^)	-	<0.1	0.1–0.3	≥0.3		
Regurgitant volume (mL)	-	<30	30–59	≥60		
Regurgitant fraction (%)	-	<30	30–49	≥50		
Diastolic flow reversal^b^	-	Brief, early	Intermediate	Holodiastolic (end-diastolic velocity >20 cm/sec)		
LVEF (%)	Normal	Normal		>50	≤50	Any
LVESD (mm)	Normal	Normal		<50	>50 (25^a^)	Dilation ↑↑
Symptom	None	None		None		DOE, Angina, HF

*BAV* Bicuspid aortic valve, *LVOT* Left ventricular outflow tract, *JCSA* Jet cross section area, *EROA* Effective regurgitant orifice area by two-dimensional proximal isovelocity surface area method, *LVEF* Left ventricular ejection fraction, *LVESD* Left ventricular endsystolic dimension, *DOE* Dyspnea on exertion, *HF* Heart failure

^a^LVESD index (mm/m^2^)

^b^Doppler flow in the descending aorta

### Korean data

A large Korean study of 4,089 patients with moderate to severe VHD, the Korean Valve Survey registry [7], was recently published. The registry reported AR in 22.6% of Korean patients (926 of 4,089) with significant VHD. The mean age of patients with AR was 70.4 ± 13.4 years, and 52.4% of them were female. Degenerative changes were identified as the most common etiology of AR, accounting for 64.3% of cases.

Another Korean study examined 23,254 asymptomatic healthy adults older than 50 years who underwent a comprehensive health checkup, and 9.4% were newly diagnosed with VHD [89]. AR was the second most commonly diagnosed VHD after tricuspid regurgitation. That study also found that at least moderate VHD was diagnosed in 0.8% of the healthy subjects, and AR was again the second most common significant VHD after tricuspid regurgitation.

Several studies have been conducted in Korea to evaluate the predictors of postoperative outcomes in patients with AR. One study showed that preoperative indexed LVESD and LVEDD were independent predictors of restoration of LV systolic function at 6 months after AVR in patients with preoperative LV systolic dysfunction (EF < 50%) or severe LV dilatation (LVEDD ≥ 70 mm or LVESD ≥ 50 mm) [101]. Kim et al. [102] reported the long-term results of 280 patients who underwent AVR for isolated AR. The 10-year survival rates were 87.3% and 80.1% in the groups with preoperative LVEF ≥ 50% and < 50%, respectively. The 10-year cardiac mortality–free survival rates in the two groups were 97.2% and 92.9%, respectively. In patients with LVEF < 50%, the preoperative E/e’ ratio was an independent predictor of all-cause mortality during follow-up.

### Diagnosis and follow-up

#### Diagnosis

TTE is the key diagnostic tool for determining the etiology, severity, and chronicity of AR, as well as for evaluating the aortic root and LV. Assessments of the morphology of the AV and aorta can identify the underlying mechanisms and feasibility of surgical treatment. AR can be classified according to changes in AV morphology, such as aortic dilatation, leaflet prolapse, restrictive valve motion, and leaflet perforation.

The aortic root and ascending aorta can be measured at the aortic annulus, sinus of Valsalva, sinotubular junction, and tubular ascending aorta. A sinus of Valsalva > 45 mm or any aortic diameter > 40 mm is considered an aortic aneurysm, which is important in the classification of AR provided above. TEE can be used to better discriminate the mechanism of AR.

The hemodynamics and cardiac adaptation in acute AR differ from those of chronic AR. In severe acute AR, the LV end-diastolic pressure might increase abruptly because the LV is not sufficiently dilated to compensate for the increase in volume. The acute increase of preload results in pulmonary edema and low forward cardiac output. In chronic AR, gradual LV dilation occurs, and the systolic function remains preserved until later stages. Therefore, echocardiography to detect changes in LV geometry and function is essential in the evaluation of chronic AR. Early detection of LV remodeling can be achieved with 2D strain imaging and 3D echocardiography [103].

For severity assessment, color Doppler, pulsed wave, and continuous wave Doppler echocardiography are necessary. The echocardiographic criteria for severe AR are the following: (1) central jet width assessed by color Doppler ≥ 65% of the width of LVOT at a Nyquist limit of 50–60 cm/sec; (2) vena contracta width > 0.6 cm; (3) regurgitant fraction ≥ 50%; (4) regurgitant volume ≥ 60 mL/beat; (5) effective regurgitant orifice area ≥ 0.30 cm^2^; and (6) presence of holodiastolic flow reversal in the proximal abdominal aorta. The continuous wave Doppler of the AR jet shows a rapid deceleration time in patients with severe AR. A pressure half-time < 300 ms on the AR velocity curve indicates rapid equalization of the aortic and LV pressures during diastole.

Cardiac CT is helpful in evaluating valve morphology and detecting aortopathy. Especially in acute AR, acute aortic dissection can be accurately detected or excluded with chest CT angiography. In patients with poor echocardiographic images, when there is discordance between clinical and echocardiographic profiles, or in patients with ambiguous valve morphology, CMR can be useful. The mechanism of AR, degree of AR severity, changes in LV geometry, and combined aortopathy can be assessed precisely with CMR [104, 105].

#### Follow-up

Reassessment of AR can be performed every 2 years in patients with mild to moderate AR. Asymptomatic patients with severe AR and normal LV function should be followed closely with serial echocardiography once a year. In patients with rapid changes in LV diameter and/or LVEF or approaching surgical standards, the follow-up interval should be shortened to 6 months. If the ascending aorta is dilated with a dimension > 40 mm and/or shows an increase > 3 mm compared with baseline, chest CT angiography or CMR should be performed.

### Medical therapy

In patients with symptomatic chronic AR, angiotensin-converting enzyme inhibitors or dihydropyridines can provide symptomatic improvement. Concomitant use of β-blockers helps to relieve symptoms in patients with heart failure after valvular surgery. The use of β-blockers should also be considered in patients with Marfan syndrome to reduce shear stress.

### Timing of intervention

**Fig. 3:**
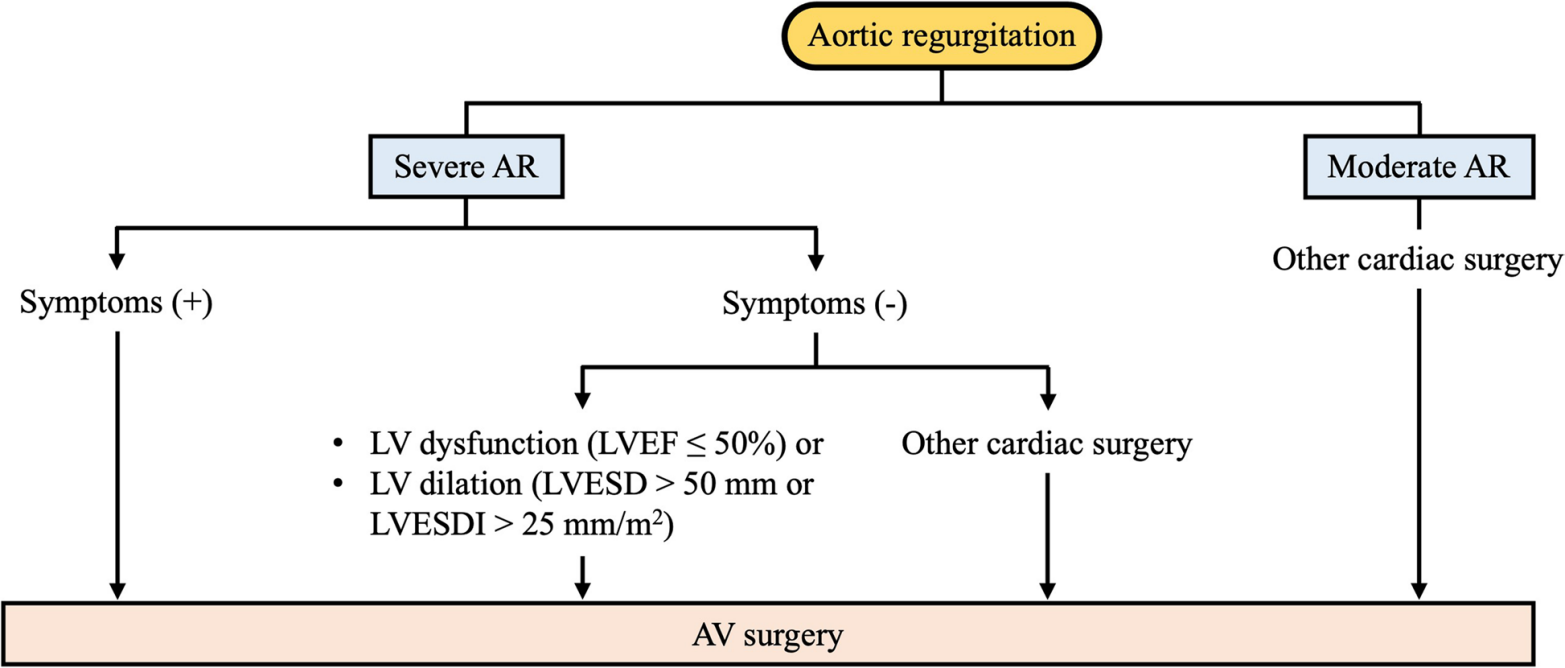
Treatment of aortic regurgitation (AR). AV, aortic valve; LV, left ventricle; LVEF, left ventricular ejection fraction; LVESD, left ventricular end-systolic dimension; LVESDI, left ventricular end-systolic dimension index

Acute AR is often difficult to treat medically because it is mainly caused by infective endocarditis, aortic dissection, or traumatic valve destruction. Therefore, urgent surgical intervention should be considered. In patients with severe chronic AR, the decision to recommend surgery is based on various factors including symptoms, LV systolic function (EF), LV dilatation, and whether any other open-heart surgery is being performed (Fig. 3). Patients with severe chronic AR who experience symptoms such as exertional dyspnea, angina, or other symptoms of heart failure are recommended to undergo surgery, regardless of their LVEF and LV cavity size, unless surgery poses a prohibitive risk or is contraindicated [106, 107]. LV systolic dysfunction and LV dilatation are strong predictors of survival and functional status after surgery for severe AR. The suggested cutoff for LV systolic dysfunction is an LVEF ≤ 50% [102, 108–111] when other causes of LV dysfunction can be excluded. LVESD > 50 mm was considered a reasonable value to determine the timing of surgery in studies performed in Western societies [110, 112, 113]. However, studies in Korea and other Asian countries have reported a smaller cutoff value, between 45 and 50 mm, as reasonable to predict postoperative prognosis [114, 115]. Most previous studies have used unadjusted LVESD, but a recent study reported that indexed LVESD (LVESD corrected for body surface area) can predict prognosis more accurately than unadjusted LVESD, especially in patients with a body surface area less than 1.68 m^2^ [116]. Indexed LVESD might be useful in determining the timing of surgery, considering the small body size typical of Korean and other Asian populations. The suggested cutoff value of indexed LVESD for optimal postoperative outcomes is 25 mm/m^2^ [82, 84, 116].

### Choice of intervention

The choice of surgical procedure should be based on the surgeon’s experience, the presence of aortic root dilatation, valve morphology, life expectancy, and the potential need for anticoagulation. AVR is the standard surgical procedure for most patients with severe AR. In experienced centers, AV repair can be considered in the small group of selected patients with favorable valve anatomy [117–119]. The advantages of AV repair include the possibility of preserving normal valve morphology and the low thromboembolic risk. However, AV repair is not widely used because of its low long-term success rate. Young patients with AR caused by aortic dilatation without valve thickening, calcification, or deformity might be candidates for valve-sparing aortic replacement [117, 120, 121]. In patients indicated for surgery for severe AR, concomitant aortic surgery is considered for a maximal ascending aortic diameter ≥ 45 mm.

## Conclusions

As the aged population grows, so does the prevalence of AV disease, particularly from degenerative causes. Echocardiography is vital in the diagnosis and severity assessment of AV disease, determining treatment strategy, and post-treatment follow-up, and multimodality imaging is often useful. Data on expanded patient population and long-term outcomes of novel therapies such as TAVR, and the therapeutic impact of early intervention will be incorporated into future revised guidelines.

## Data Availability

Not applicable.

## Abbreviations

ACC/AHA: American College of Cardiology/American Heart Association
AR: Aortic regurgitation
AS: Aortic stenosis
AV: Aortic valve
AVA: Aortic valve area
AVR: Aortic valve replacement
BAV: Bicuspid aortic valve
CI: Confidence interval
CMR: Cardiac magnetic resonance
CT: Computed tomography
D: Dimensional
EF: Ejection fraction
ESC/EACTS: European Society of Cardiology/European Association of Cardio-Thoracic Surgeons
HFrEF: Heart failure with reduced ejection fraction
HR: Hazard ratio
KSE: Korean Society of Echocardiography
LV: Left ventricular
LVEDD: Left ventricular end-diastolic dimension
LVEF: Left ventricular ejection fraction
LVESD: Left ventricular end-systolic dimension
LVOT: Left ventricular outflow tract
MG: Mean pressure gradient
RCT: Randomized controlled trial
SAVR: Surgical aortic valve replacement
SV: Stroke volume
SVi: Stroke volume indexed by body surface area
TAVR: Transcatheter aortic valve replacement
TEE: Transesophageal echocardiography
TTE: Transthoracic echocardiography
VHD: Valvular heart disease
Vmax: Peak aortic jet velocity

## References

[1] Otto CM, Nishimura RA, Bonow RO, Carabello BA, Erwin JP, Gentile F, et al. 2020 ACC/AHA guideline for the management of patients with valvular heart disease: a report of the American College of Cardiology/American Heart Association joint committee on clinical practice guidelines. Circulation. 2021;143:e72-227. 33332150 10.1161/CIR.0000000000000923

[2] Vahanian A, Beyersdorf F, Praz F, Milojevic M, Baldus S, Bauersachs J, et al. 2021 ESC/EACTS guidelines for the management of valvular heart disease. Eur Heart J. 2022;43:561–632. 34453165 10.1093/eurheartj/ehab395

[3] Iung B, Delgado V, Rosenhek R, Price S, Prendergast B, Wendler O, et al. Contemporary presentation and management of valvular heart disease: the EURObservational Research Programme valvular heart disease II survey. Circulation. 2019;140:1156–69. 31510787 10.1161/CIRCULATIONAHA.119.041080

[4] Yadgir S, Johnson CO, Aboyans V, Adebayo OM, Adedoyin RA, Afarideh M, et al. Global, regional, and national burden of calcific aortic valve and degenerative mitral valve diseases, 1990–2017. Circulation. 2020;141:1670–80. 32223336 10.1161/CIRCULATIONAHA.119.043391

[5] d’Arcy JL, Coffey S, Loudon MA, Kennedy A, Pearson-Stuttard J, Birks J, et al. Large-scale community echocardiographic screening reveals a major burden of undiagnosed valvular heart disease in older people: the OxVALVE Population Cohort Study. Eur Heart J. 2016;37:3515–22. 27354049 10.1093/eurheartj/ehw229 PMC5216199

[6] Masri A, Svensson LG, Griffin BP, Desai MY. Contemporary natural history of bicuspid aortic valve disease: a systematic review. Heart. 2017;103:1323–30. 28490615 10.1136/heartjnl-2016-309916

[7] Choi YJ, Son JW, Kim EK, Kim IC, Kim HY, Seo JS, et al. Epidemiologic profile of patients with valvular heart disease in Korea: a nationwide hospital-based registry study. J Cardiovasc Imaging. 2023;31:51–61. 36693346 10.4250/jcvi.2022.0076 PMC9880350

[8] Kang DH, Park SJ, Rim JH, Yun SC, Kim DH, Song JM, et al. Early surgery versus conventional treatment in asymptomatic very severe aortic stenosis. Circulation. 2010;121:1502–9. 20308614 10.1161/CIRCULATIONAHA.109.909903

[9] Kang DH, Park SJ, Lee SA, Lee S, Kim DH, Kim HK, et al. Early surgery or conservative care for asymptomatic aortic stenosis. N Engl J Med. 2020;382:111–9. 31733181 10.1056/NEJMoa1912846

[10] Baumgartner H, Hung J, Bermejo J, Chambers JB, Edvardsen T, Goldstein S, et al. Recommendations on the echocardiographic assessment of aortic valve stenosis: a focused update from the European Association of Cardiovascular Imaging and the American Society of Echocardiography. J Am Soc Echocardiogr. 2017;30:372–92. 28385280 10.1016/j.echo.2017.02.009

[11] Tastet L, Tribouilloy C, Maréchaux S, Vollema EM, Delgado V, Salaun E, et al. Staging cardiac damage in patients with asymptomatic aortic valve stenosis. J Am Coll Cardiol. 2019;74:550–63. 31345430 10.1016/j.jacc.2019.04.065

[12] Otto CM, Prendergast B. Aortic-valve stenosis: from patients at risk to severe valve obstruction. N Engl J Med. 2014;371:744–56. 25140960 10.1056/NEJMra1313875

[13] Remenyi B, ElGuindy A, Smith SC, Yacoub M, Holmes DR. Valvular aspects of rheumatic heart disease. Lancet. 2016;387:1335–46. 27025439 10.1016/S0140-6736(16)00547-X

[14] Hachicha Z, Dumesnil JG, Bogaty P, Pibarot P. Paradoxical low-flow, low-gradient severe aortic stenosis despite preserved ejection fraction is associated with higher afterload and reduced survival. Circulation. 2007;115:2856–64. 17533183 10.1161/CIRCULATIONAHA.106.668681

[15] Pibarot P, Dumesnil JG. Improving assessment of aortic stenosis. J Am Coll Cardiol. 2012;60:169–80. 22789881 10.1016/j.jacc.2011.11.078

[16] Briand M, Dumesnil JG, Kadem L, Tongue AG, Rieu R, Garcia D, et al. Reduced systemic arterial compliance impacts significantly on left ventricular afterload and function in aortic stenosis: implications for diagnosis and treatment. J Am Coll Cardiol. 2005;46:291–8. 16022957 10.1016/j.jacc.2004.10.081

[17] Clavel MA, Dumesnil JG, Capoulade R, Mathieu P, Senechal M, Pibarot P. Outcome of patients with aortic stenosis, small valve area, and low-flow, low-gradient despite preserved left ventricular ejection fraction. J Am Coll Cardiol. 2012;60:1259–67. 22657269 10.1016/j.jacc.2011.12.054

[18] Minners J, Allgeier M, Gohlke-Baerwolf C, Kienzle RP, Neumann FJ, Jander N. Inconsistencies of echocardiographic criteria for the grading of aortic valve stenosis. Eur Heart J. 2008;29:1043–8. 18156619 10.1093/eurheartj/ehm543

[19] Clavel MA, Fuchs C, Burwash IG, Mundigler G, Dumesnil JG, Baumgartner H, et al. Predictors of outcomes in low-flow, low-gradient aortic stenosis: results of the multicenter TOPAS Study. Circulation. 2008;118:S234–42. 18824760 10.1161/CIRCULATIONAHA.107.757427

[20] Nishimura RA, Grantham JA, Connolly HM, Schaff HV, Higano ST, Holmes DR Jr. Low-output, low-gradient aortic stenosis in patients with depressed left ventricular systolic function: the clinical utility of the dobutamine challenge in the catheterization laboratory. Circulation. 2002;106:809–13. 12176952 10.1161/01.cir.0000025611.21140.34

[21] Pibarot P, Dumesnil JG. Low-flow, low-gradient aortic stenosis with normal and depressed left ventricular ejection fraction. J Am Coll Cardiol. 2012;60:1845–53. 23062546 10.1016/j.jacc.2012.06.051

[22] Jander N, Minners J, Holme I, Gerdts E, Boman K, Brudi P, et al. Outcome of patients with low-gradient “severe” aortic stenosis and preserved ejection fraction. Circulation. 2011;123:887–95. 21321152 10.1161/CIRCULATIONAHA.110.983510

[23] Eleid MF, Sorajja P, Michelena HI, Malouf JF, Scott CG, Pellikka PA. Survival by stroke volume index in patients with low-gradient normal EF severe aortic stenosis. Heart. 2015;101:23–9. 25217490 10.1136/heartjnl-2014-306151

[24] Lee PH, Hong JA, Sun BJ, Han S, Park S, Jang JY, et al. Impact of significant mitral regurgitation on assessing the severity of aortic stenosis. J Am Soc Echocardiogr. 2018;31:26–33. 29158019 10.1016/j.echo.2017.09.012

[25] Mehrotra P, Jansen K, Flynn AW, Tan TC, Elmariah S, Picard MH, et al. Differential left ventricular remodelling and longitudinal function distinguishes low flow from normal-flow preserved ejection fraction low-gradient severe aortic stenosis. Eur Heart J. 2013;34:1906–14. 23533186 10.1093/eurheartj/eht094 PMC3858103

[26] Rusinaru D, Malaquin D, Marechaux S, Debry N, Tribouilloy C. Relation of dimensionless index to long-term outcome in aortic stenosis with preserved LVEF. JACC Cardiovasc Imaging. 2015;8:766–75. 26093931 10.1016/j.jcmg.2015.01.023

[27] Vollema EM, Sugimoto T, Shen M, Tastet L, Ng ACT, Abou R, et al. Association of left ventricular global longitudinal strain with asymptomatic severe aortic stenosis: natural course and prognostic value. JAMA Cardiol. 2018;3:839–47. 30140889 10.1001/jamacardio.2018.2288 PMC6233650

[28] Magne J, Cosyns B, Popescu BA, Carstensen HG, Dahl J, Desai MY, et al. Distribution and prognostic significance of left ventricular global longitudinal strain in asymptomatic significant aortic stenosis: an individual participant data meta-analysis. JACC Cardiovasc Imaging. 2019;12:84–92. 30621997 10.1016/j.jcmg.2018.11.005

[29] Bergler-Klein J, Klaar U, Heger M, Rosenhek R, Mundigler G, Gabriel H, et al. Natriuretic peptides predict symptom-free survival and postoperative outcome in severe aortic stenosis. Circulation. 2004;109:2302–8. 15117847 10.1161/01.CIR.0000126825.50903.18

[30] Clavel MA, Malouf J, Michelena HI, Suri RM, Jaffe AS, Mahoney DW, et al. B-type natriuretic peptide clinical activation in aortic stenosis: impact on long-term survival. J Am Coll Cardiol. 2014;63:2016–25. 24657652 10.1016/j.jacc.2014.02.581

[31] Maréchaux S, Hachicha Z, Bellouin A, Dumesnil JG, Meimoun P, Pasquet A, et al. Usefulness of exercise-stress echocardiography for risk stratification of true asymptomatic patients with aortic valve stenosis. Eur Heart J. 2010;31:1390–7. 20308041 10.1093/eurheartj/ehq076 PMC2878968

[32] Saeed S, Rajani R, Seifert R, Parkin D, Chambers JB. Exercise testing in patients with asymptomatic moderate or severe aortic stenosis. Heart. 2018;104:1836–42. 29654094 10.1136/heartjnl-2018-312939 PMC6241614

[33] Atterhog JH, Jonsson B, Samuelsson R. Exercise testing: a prospective study of complication rates. Am Heart J. 1979;98:572–9. 495403 10.1016/0002-8703(79)90282-5

[34] Clavel MA, Pibarot P, Messika-Zeitoun D, Capoulade R, Malouf J, Aggarval S, et al. Impact of aortic valve calcification, as measured by MDCT, on survival in patients with aortic stenosis: results of an international registry study. J Am Coll Cardiol. 2014;64:1202–13. 25236511 10.1016/j.jacc.2014.05.066 PMC4391203

[35] Pawade T, Clavel MA, Tribouilloy C, Dreyfus J, Mathieu T, Tastet L, et al. Computed tomography aortic valve calcium scoring in patients with aortic stenosis. Circ Cardiovasc Imaging. 2018;11:e007146. 29555836 10.1161/CIRCIMAGING.117.007146

[36] Borger MA, Fedak PW, Stephens EH, Gleason TG, Girdauskas E, Ikonomidis JS, et al. The American Association for Thoracic Surgery consensus guidelines on bicuspid aortic valve-related aortopathy: full online-only version. J Thorac Cardiovasc Surg. 2018;156:e41-74. 30011777 10.1016/j.jtcvs.2018.02.115 PMC6413866

[37] Everett RJ, Treibel TA, Fukui M, Lee H, Rigolli M, Singh A, et al. Extracellular myocardial volume in patients with aortic stenosis. J Am Coll Cardiol. 2020;75:304–16. 31976869 10.1016/j.jacc.2019.11.032 PMC6985897

[38] Nitsche C, Scully PR, Patel KP, Kammerlander AA, Koschutnik M, Dona C, et al. Prevalence and outcomes of concomitant aortic stenosis and cardiac amyloidosis. J Am Coll Cardiol. 2021;77:128–39. 33181246 10.1016/j.jacc.2020.11.006 PMC7805267

[39] Park SJ, Cho SW, Kim SM, Ahn J, Carriere K, Jeong DS, et al. Assessment of myocardial fibrosis using multimodality imaging in severe aortic stenosis: comparison with histologic fibrosis. JACC Cardiovasc Imaging. 2019;12:109–19. 30448148 10.1016/j.jcmg.2018.05.028

[40] Kwak S, Everett RJ, Treibel TA, Yang S, Hwang D, Ko T, et al. Markers of myocardial damage predict mortality in patients with aortic stenosis. J Am Coll Cardiol. 2021;78:545–58. 34353531 10.1016/j.jacc.2021.05.047

[41] Nishimura RA, Carabello BA. Hemodynamics in the cardiac catheterization laboratory of the 21st century. Circulation. 2012;125:2138–50. 22547754 10.1161/CIRCULATIONAHA.111.060319

[42] Dekany G, Fontos G, Satish S, Szabo G, Pinter T, Piroth Z, et al. The prognostic value of immediate post-TAVI hemodynamic evaluation is superior to aortography and transoesophageal echocardiography in predicting patient survival. Int J Cardiol. 2021;329:153–61. 33359335 10.1016/j.ijcard.2020.12.058

[43] Cosmi JE, Kort S, Tunick PA, Rosenzweig BP, Freedberg RS, Katz ES, et al. The risk of the development of aortic stenosis in patients with “benign” aortic valve thickening. Arch Intern Med. 2002;162:2345–7. 12418948 10.1001/archinte.162.20.2345

[44] Rahimi K, Mohseni H, Kiran A, Tran J, Nazarzadeh M, Rahimian F, et al. Elevated blood pressure and risk of aortic valve disease: a cohort analysis of 5.4 million UK adults. Eur Heart J. 2018;39:3596–603. 30212891 10.1093/eurheartj/ehy486 PMC6186276

[45] Nielsen OW, Sajadieh A, Sabbah M, Greve AM, Olsen MH, Boman K, et al. Assessing optimal blood pressure in patients with asymptomatic aortic valve stenosis: the simvastatin ezetimibe in aortic stenosis study (SEAS). Circulation. 2016;134:455–68. 27486164 10.1161/CIRCULATIONAHA.115.021213

[46] Rossebø AB, Pedersen TR, Boman K, Brudi P, Chambers JB, Egstrup K, et al. Intensive lipid lowering with simvastatin and ezetimibe in aortic stenosis. N Engl J Med. 2008;359:1343–56. 18765433 10.1056/NEJMoa0804602

[47] Cowell SJ, Newby DE, Prescott RJ, Bloomfield P, Reid J, Northridge DB, et al. A randomized trial of intensive lipid-lowering therapy in calcific aortic stenosis. N Engl J Med. 2005;352:2389–97. 15944423 10.1056/NEJMoa043876

[48] Chan KL, Teo K, Dumesnil JG, Ni A, Tam J, ASTRONOMER Investigators. Effect of lipid lowering with rosuvastatin on progression of aortic stenosis: results of the aortic stenosis progression observation: measuring effects of rosuvastatin (ASTRONOMER) trial. Circulation. 2010;121:306–14. 20048204 10.1161/CIRCULATIONAHA.109.900027

[49] Lee W, Choi W, Kang SH, Hwang IC, Choi HM, Yoon YE, et al. Long-term prognosis of mild to moderate aortic stenosis and coronary artery disease. J Korean Med Sci. 2021;36:e47. 33559407 10.3346/jkms.2021.36.e47 PMC7870422

[50] Inohara T, Manandhar P, Kosinski AS, Matsouaka RA, Kohsaka S, Mentz RJ, et al. Association of renin-angiotensin inhibitor treatment with mortality and heart failure readmission in patients with transcatheter aortic valve replacement. JAMA. 2018;320:2231–41. 30512100 10.1001/jama.2018.18077 PMC6583475

[51] Ochiai T, Saito S, Yamanaka F, Shishido K, Tanaka Y, Yamabe T, et al. Renin-angiotensin system blockade therapy after transcatheter aortic valve implantation. Heart. 2018;104:644–51. 28986405 10.1136/heartjnl-2017-311738

[52] Pawade TA, Doris MK, Bing R, White AC, Forsyth L, Evans E, et al. Effect of denosumab or alendronic acid on the progression of aortic stenosis: a double-blind randomized controlled trial. Circulation. 2021;143:2418–27. 33913339 10.1161/CIRCULATIONAHA.121.053708 PMC8212878

[53] Lee S, Lee SA, Choi B, Kim YJ, Oh SJ, Choi HM, et al. Dipeptidyl peptidase-4 inhibition to prevent progression of calcific aortic stenosis. Heart. 2020;106:1824–31. 32917732 10.1136/heartjnl-2020-317024 PMC7677484

[54] Kumar A, Majmundar M, Doshi R, Kansara T, Shariff M, Shah P, et al. Meta-analysis of early intervention versus conservative management for asymptomatic severe aortic stenosis. Am J Cardiol. 2021;138:85–91. 33065088 10.1016/j.amjcard.2020.10.013

[55] Gahl B, Çelik M, Head SJ, Vanoverschelde JL, Pibarot P, Reardon MJ, et al. Natural history of asymptomatic severe aortic stenosis and the association of early intervention with outcomes: a systematic review and meta-analysis. JAMA Cardiol. 2020;5:1102–12. 32639521 10.1001/jamacardio.2020.2497 PMC7344834

[56] Wojnarski CM. Commentary: evidence is mounting for early intervention in asymptomatic severe aortic stenosis. J Thorac Cardiovasc Surg. 2022;163:1790–1. 32980148 10.1016/j.jtcvs.2020.08.078

[57] Ennezat PV, Malergue MC, Le Jemtel TH, Abergel E. Watchful waiting care or early intervention in asymptomatic severe aortic stenosis: where we are. Arch Cardiovasc Dis. 2021;114:59–72. 33153947 10.1016/j.acvd.2020.07.002

[58] Banovic M, Putnik S, Penicka M, Doros G, Deja MA, Kockova R, et al. Aortic valve replacement versus conservative treatment in asymptomatic severe aortic stenosis: the AVATAR trial. Circulation. 2022;145:648–58. 34779220 10.1161/CIRCULATIONAHA.121.057639

[59] Bing R, Everett RJ, Tuck C, Semple S, Lewis S, Harkess R, et al. Rationale and design of the randomized, controlled Early Valve Replacement Guided by Biomarkers of Left Ventricular Decompensation in Asymptomatic Patients with Severe Aortic Stenosis (EVOLVED) trial. Am Heart J. 2019;212:91–100. 30978556 10.1016/j.ahj.2019.02.018

[60] Badiani S, Bhattacharyya S, Aziminia N, Treibel TA, Lloyd G. Moderate aortic stenosis: what is it and when should we intervene? Interv Cardiol. 2021;16:e09. 34188693 10.15420/icr.2021.04 PMC8201468

[61] Everett RJ, Clavel MA, Pibarot P, Dweck MR. Timing of intervention in aortic stenosis: a review of current and future strategies. Heart. 2018;104:2067–76. 30030337 10.1136/heartjnl-2017-312304 PMC6287563

[62] Lancellotti P, Vannan MA. Timing of intervention in aortic stenosis. N Engl J Med. 2020;382:191–3. 31733179 10.1056/NEJMe1914382

[63] Ito S, Miranda WR, Nkomo VT, Boler AN, Pislaru SV, Pellikka PA, et al. Prognostic risk stratification of patients with moderate aortic stenosis. J Am Soc Echocardiogr. 2021;34:248–56. 33161066 10.1016/j.echo.2020.10.012

[64] Jean G, Van Mieghem NM, Gegenava T, van Gils L, Bernard J, Geleijnse ML, et al. Moderate aortic stenosis in patients with heart failure and reduced ejection fraction. J Am Coll Cardiol. 2021;77:2796–803. 34082909 10.1016/S0735-1097(21)04151-6 PMC8091313

[65] Cormican DS, Czerny M, Ramakrishna H. The dilemma of moderate aortic stenosis in patients with heart failure and reduced ejection fraction: are we waiting too long to intervene, or should we wait for more evidence? J Cardiothorac Vasc Anesth. 2022;36:15–7. 34518100 10.1053/j.jvca.2021.08.024

[66] Chikwe J, Chiang YP, Egorova NN, Itagaki S, Adams DH. Survival and outcomes following bioprosthetic vs mechanical mitral valve replacement in patients aged 50 to 69 years. JAMA. 2015;313:1435–42. 25871669 10.1001/jama.2015.3164

[67] McClure RS, McGurk S, Cevasco M, Maloney A, Gosev I, Wiegerinck EM, et al. Late outcomes comparison of nonelderly patients with stented bioprosthetic and mechanical valves in the aortic position: a propensity-matched analysis. J Thorac Cardiovasc Surg. 2014;148:1931–9. 24521965 10.1016/j.jtcvs.2013.12.042

[68] Chiang YP, Chikwe J, Moskowitz AJ, Itagaki S, Adams DH, Egorova NN. Survival and long-term outcomes following bioprosthetic vs mechanical aortic valve replacement in patients aged 50 to 69 years. JAMA. 2014;312:1323–9. 25268439 10.1001/jama.2014.12679

[69] Kaneko T, Aranki S, Javed Q, McGurk S, Shekar P, Davidson M, et al. Mechanical versus bioprosthetic mitral valve replacement in patients <65 years old. J Thorac Cardiovasc Surg. 2014;147:117–26. 24079878 10.1016/j.jtcvs.2013.08.028

[70] Buratto E, Shi WY, Wynne R, Poh CL, Larobina M, O’Keefe M, et al. Improved survival after the ross procedure compared with mechanical aortic valve replacement. J Am Coll Cardiol. 2018;71:1337–44. 29566818 10.1016/j.jacc.2018.01.048

[71] Tuzcu EM, Kapadia SR, Vemulapalli S, Carroll JD, Holmes DR, Mack MJ, et al. Transcatheter aortic valve replacement of failed surgically implanted bioprostheses: the STS/ACC registry. J Am Coll Cardiol. 2018;72:370–82. 30025572 10.1016/j.jacc.2018.04.074

[72] Ye J, Cheung A, Yamashita M, Wood D, Peng D, Gao M, et al. Transcatheter aortic and mitral valve-in-valve implantation for failed surgical bioprosthetic valves: an 8-year single-center experience. JACC Cardiovasc Interv. 2015;8:1735–44. 26476608 10.1016/j.jcin.2015.08.012

[73] Dvir D, Webb JG, Bleiziffer S, Pasic M, Waksman R, Kodali S, et al. Transcatheter aortic valve implantation in failed bioprosthetic surgical valves. JAMA. 2014;312:162–70. 25005653 10.1001/jama.2014.7246

[74] Leon MB, Smith CR, Mack M, Miller DC, Moses JW, Svensson LG, et al. Transcatheter aortic-valve implantation for aortic stenosis in patients who cannot undergo surgery. N Engl J Med. 2010;363:1597–607. 20961243 10.1056/NEJMoa1008232

[75] Smith CR, Leon MB, Mack MJ, Miller DC, Moses JW, Svensson LG, et al. Transcatheter versus surgical aortic-valve replacement in high-risk patients. N Engl J Med. 2011;364:2187–98. 21639811 10.1056/NEJMoa1103510

[76] Leon MB, Smith CR, Mack MJ, Makkar RR, Svensson LG, Kodali SK, et al. Transcatheter or surgical aortic-valve replacement in intermediate-risk patients. N Engl J Med. 2016;374:1609–20. 27040324 10.1056/NEJMoa1514616

[77] Mack MJ, Leon MB, Thourani VH, Makkar R, Kodali SK, Russo M, et al. Transcatheter aortic-valve replacement with a balloon-expandable valve in low-risk patients. N Engl J Med. 2019;380:1695–705. 30883058 10.1056/NEJMoa1814052

[78] Fu J, Popal MS, Li Y, Li G, Qi Y, Fang F, et al. Transcatheter versus surgical aortic valve replacement in low and intermediate risk patients with severe aortic stenosis: systematic review and meta-analysis of randomized controlled trials and propensity score matching observational studies. J Thorac Dis. 2019;11:1945–62. 31285888 10.21037/jtd.2019.04.97 PMC6588740

[79] Reardon MJ, Van Mieghem NM, Popma JJ, Kleiman NS, Søndergaard L, Mumtaz M, et al. Surgical or transcatheter aortic-valve replacement in intermediate-risk patients. N Engl J Med. 2017;376:1321–31. 28304219 10.1056/NEJMoa1700456

[80] Thyregod HG, Steinbrüchel DA, Ihlemann N, Nissen H, Kjeldsen BJ, Petursson P, et al. Transcatheter versus surgical aortic valve replacement in patients with severe aortic valve stenosis: 1-year results from the all-comers NOTION randomized clinical trial. J Am Coll Cardiol. 2015;65:2184–94. 25787196 10.1016/j.jacc.2015.03.014

[81] Jørgensen TH, Thyregod HGH, Ihlemann N, Nissen H, Petursson P, Kjeldsen BJ, et al. Eight-year outcomes for patients with aortic valve stenosis at low surgical risk randomized to transcatheter vs. surgical aortic valve replacement. Eur Heart J. 2021;42:2912–9. 34179981 10.1093/eurheartj/ehab375 PMC8347457

[82] Mentias A, Feng K, Alashi A, Rodriguez LL, Gillinov AM, Johnston DR, et al. Long-term outcomes in patients with aortic regurgitation and preserved left ventricular ejection fraction. J Am Coll Cardiol. 2016;68:2144–53. 27855803 10.1016/j.jacc.2016.08.045

[83] Yang LT, Michelena HI, Scott CG, Enriquez-Sarano M, Pislaru SV, Schaff HV, et al. Outcomes in chronic hemodynamically significant aortic regurgitation and limitations of current guidelines. J Am Coll Cardiol. 2019;73:1741–52. 30846339 10.1016/j.jacc.2019.01.024

[84] de Meester C, Gerber BL, Vancraeynest D, Pouleur AC, Noirhomme P, Pasquet A, et al. Do guideline-based indications result in an outcome penalty for patients with severe aortic regurgitation? JACC Cardiovasc Imaging. 2019;12:2126–38. 30660551 10.1016/j.jcmg.2018.11.022

[85] Yang LT, Enriquez-Sarano M, Michelena HI, Nkomo VT, Scott CG, Bailey KR, et al. Predictors of progression in patients with stage B aortic regurgitation. J Am Coll Cardiol. 2019;74:2480–92. 31727286 10.1016/j.jacc.2019.08.1058

[86] Yoon SH, Bleiziffer S, De Backer O, Delgado V, Arai T, Ziegelmueller J, et al. Outcomes in transcatheter aortic valve replacement for bicuspid versus tricuspid aortic valve stenosis. J Am Coll Cardiol. 2017;69:2579–89. 28330793 10.1016/j.jacc.2017.03.017

[87] Forrest JK, Kaple RK, Ramlawi B, Gleason TG, Meduri CU, Yakubov SJ, et al. Transcatheter aortic valve replacement in bicuspid versus tricuspid aortic valves from the STS/ACC TVT registry. JACC Cardiovasc Interv. 2020;13:1749–59. 32473890 10.1016/j.jcin.2020.03.022

[88] Song S, Seo J, Cho I, Hong GR, Ha JW, Shim CY. Progression and outcomes of non-dysfunctional bicuspid aortic valve: longitudinal data from a large Korean bicuspid aortic valve registry. Front Cardiovasc Med. 2020;7:603323. 33505993 10.3389/fcvm.2020.603323 PMC7829218

[89] Kim MS, Cho SJ, Park SJ, Cho SW, Choi SH, Kim HS, et al. Frequency and clinical associating factors of valvular heart disease in asymptomatic Korean adults. Sci Rep. 2019;9:16741. 31727975 10.1038/s41598-019-53277-0 PMC6856181

[90] Sun BJ, Jin X, Song JK, Lee S, Lee JH, Park JB, et al. Clinical characteristics of Korean patients with bicuspid aortic valve who underwent aortic valve surgery. Korean Circ J. 2018;48:48–58. 29171200 10.4070/kcj.2017.0124 PMC5764870

[91] Jeong H, Shim CY, Kim D, Choi JY, Choi KU, Lee SY, et al. Prevalence, characteristics, and clinical significance of concomitant cardiomyopathies in subjects with bicuspid aortic valves. Yonsei Med J. 2019;60:816–23. 31433579 10.3349/ymj.2019.60.9.816 PMC6704018

[92] Lee SY, Shim CY, Kim D, Cho I, Hong GR, Ha JW, et al. Factors determining aortic valve dysfunction in Korean subjects with a bicuspid aortic valve. Am J Cardiol. 2017;119:2049–55. 28434646 10.1016/j.amjcard.2017.03.038

[93] Yu CW, Kim WJ, Ahn JM, Kook H, Kang SH, Han JK, et al. Trends and outcomes of transcatheter aortic valve implantation (TAVI) in Korea: the results of the first cohort of Korean TAVI registry. Korean Circ J. 2018;48:382–94. 29671283 10.4070/kcj.2018.0117 PMC5940643

[94] Kim H, Lee SJ, Hong SJ, Shim CY, Ahn CM, Kim JS, et al. Clinical outcomes of transcatheter aortic valve implantation for native aortic valves in patients with low coronary heights. Yonsei Med J. 2021;62:209–14. 33635010 10.3349/ymj.2021.62.3.209 PMC7934105

[95] Lee CH, Ko YG, Park Y, Shim CY, Hong GR, Lee SH, et al. Risk factors for closure failure following percutaneous transfemoral transcatheter aortic valve implantation. Ann Vasc Surg. 2020;66:406–14. 31918036 10.1016/j.avsg.2019.12.034

[96] Kim C, Hong MK. Aortic stenosis and transcatheter aortic valve implantation: current status and future directions in Korea. Korean Circ J. 2019;49:283–97. 30895756 10.4070/kcj.2019.0044 PMC6428950

[97] Kim C, Hong MK. Good patients make favorable clinical outcome: K-TAVI registry reports. Korean Circ J. 2018;48:427–9. 29737641 10.4070/kcj.2018.0095 PMC5940647

[98] Iung B, Vahanian A. Epidemiology of acquired valvular heart disease. Can J Cardiol. 2014;30:962–70. 24986049 10.1016/j.cjca.2014.03.022

[99] Maurer G. Aortic regurgitation. Heart. 2006;92:994–1000. 16775114 10.1136/hrt.2004.042614 PMC1860728

[100] Flint N, Wunderlich NC, Shmueli H, Ben-Zekry S, Siegel RJ, Beigel R. Aortic regurgitation. Curr Cardiol Rep. 2019;21:65. 31161305 10.1007/s11886-019-1144-6

[101] Cho SH, Byun CS, Kim KW, Chang BC, Yoo KJ, Lee S. Preoperative indexed left ventricular dimensions to predict early recovery of left ventricular function after aortic valve replacement for chronic aortic regurgitation. Circ J. 2010;74:2340–5. 20921816 10.1253/circj.cj-10-0278

[102] Kim MS, Kim JH, Joo HC, Lee S, Youn YN, Lee SH. Prognostic markers and long-term outcomes after aortic valve replacement in patients with chronic aortic regurgitation. J Am Heart Assoc. 2020;9:e018292. 33289450 10.1161/JAHA.120.018292 PMC7955401

[103] Medvedofsky D, Maffessanti F, Weinert L, Tehrani DM, Narang A, Addetia K, et al. 2D and 3D echocardiography-derived indices of left ventricular function and shape: relationship with mortality. JACC Cardiovasc Imaging. 2018;11:1569–79. 29153577 10.1016/j.jcmg.2017.08.023 PMC5945352

[104] Myerson SG, d’Arcy J, Mohiaddin R, Greenwood JP, Karamitsos TD, Francis JM, et al. Aortic regurgitation quantification using cardiovascular magnetic resonance: association with clinical outcome. Circulation. 2012;126:1452–60. 22879371 10.1161/CIRCULATIONAHA.111.083600

[105] Goffinet C, Kersten V, Pouleur AC, le Polain de Waroux JB, Vancraeynest D, Pasquet A, et al. Comprehensive assessment of the severity and mechanism of aortic regurgitation using multidetector CT and MR. Eur Radiol. 2010;20:326–36. 19652976 10.1007/s00330-009-1544-x

[106] Klodas E, Enriquez-Sarano M, Tajik AJ, Mullany CJ, Bailey KR, Seward JB. Optimizing timing of surgical correction in patients with severe aortic regurgitation: role of symptoms. J Am Coll Cardiol. 1997;30:746–52. 9283535 10.1016/s0735-1097(97)00205-2

[107] Dujardin KS, Enriquez-Sarano M, Schaff HV, Bailey KR, Seward JB, Tajik AJ. Mortality and morbidity of aortic regurgitation in clinical practice A long-term follow-up study. Circulation. 1999;99:1851–7. 10199882 10.1161/01.cir.99.14.1851

[108] Chaliki HP, Mohty D, Avierinos JF, Scott CG, Schaff HV, Tajik AJ, et al. Outcomes after aortic valve replacement in patients with severe aortic regurgitation and markedly reduced left ventricular function. Circulation. 2002;106:2687–93. 12438294 10.1161/01.cir.0000038498.59829.38

[109] Kaneko T, Ejiofor JI, Neely RC, McGurk S, Ivkovic V, Stevenson LW, et al. Aortic regurgitation with markedly reduced left ventricular function is not a contraindication for aortic valve replacement. Ann Thorac Surg. 2016;102:41–7. 27016840 10.1016/j.athoracsur.2015.12.068

[110] Tornos P, Sambola A, Permanyer-Miralda G, Evangelista A, Gomez Z, Soler-Soler J. Long-term outcome of surgically treated aortic regurgitation: influence of guideline adherence toward early surgery. J Am Coll Cardiol. 2006;47:1012–7. 16516086 10.1016/j.jacc.2005.10.049

[111] Forman R, Firth BG, Barnard MS. Prognostic significance of preoperative left ventricular ejection fraction and valve lesion in patients with aortic valve replacement. Am J Cardiol. 1980;45:1120–5. 7377109 10.1016/0002-9149(80)90468-3

[112] Tornos MP, Olona M, Permanyer-Miralda G, Herrejon MP, Camprecios M, Evangelista A, et al. Clinical outcome of severe asymptomatic chronic aortic regurgitation: a long-term prospective follow-up study. Am Heart J. 1995;130:333–9. 7631617 10.1016/0002-8703(95)90450-6

[113] Turk R, Varadarajan P, Kamath A, Sampat U, Khandhar S, Patel R, et al. Survival benefit of aortic valve replacement in older patients with asymptomatic chronic severe aortic regurgitation. Ann Thorac Surg. 2010;89:731–7. 20172118 10.1016/j.athoracsur.2009.12.008

[114] Park HW, Song JM, Choo SJ, Chung CH, Lee JW, Kim DH, et al. Effect of preoperative ejection fraction, left ventricular systolic dimension and hemoglobin level on survival after aortic valve surgery in patients with severe chronic aortic regurgitation. Am J Cardiol. 2012;109:1782–6. 22459298 10.1016/j.amjcard.2012.02.024

[115] Amano M, Izumi C, Imamura S, Onishi N, Sakamoto J, Tamaki Y, et al. Pre- and postoperative predictors of long-term prognosis after aortic valve replacement for severe chronic aortic regurgitation. Circ J. 2016;80:2460–7. 27829587 10.1253/circj.CJ-16-0782

[116] Sambola A, Tornos P, Ferreira-Gonzalez I, Evangelista A. Prognostic value of preoperative indexed end-systolic left ventricle diameter in the outcome after surgery in patients with chronic aortic regurgitation. Am Heart J. 2008;155:1114–20. 18513527 10.1016/j.ahj.2007.12.025

[117] Boodhwani M, de Kerchove L, Glineur D, Poncelet A, Rubay J, Astarci P, et al. Repair-oriented classification of aortic insufficiency: impact on surgical techniques and clinical outcomes. J Thorac Cardiovasc Surg. 2009;137:286–94. 19185138 10.1016/j.jtcvs.2008.08.054

[118] Davierwala PM, David TE, Armstrong S, Ivanov J. Aortic valve repair versus replacement in bicuspid aortic valve disease. J Heart Valve Dis. 2003;12:679–86. 14658805

[119] Minakata K, Schaff HV, Zehr KJ, Dearani JA, Daly RC, Orszulak TA, et al. Is repair of aortic valve regurgitation a safe alternative to valve replacement? J Thorac Cardiovasc Surg. 2004;127:645–53. 15001892 10.1016/j.jtcvs.2003.09.018

[120] Aicher D, Kunihara T, Abou Issa O, Brittner B, Graber S, Schafers HJ. Valve configuration determines long-term results after repair of the bicuspid aortic valve. Circulation. 2011;123:178–85. 21200006 10.1161/CIRCULATIONAHA.109.934679

[121] Pettersson GB, Crucean AC, Savage R, Halley CM, Grimm RA, Svensson LG, et al. Toward predictable repair of regurgitant aortic valves: a systematic morphology-directed approach to bicommissural repair. J Am Coll Cardiol. 2008;52:40–9. 18582633 10.1016/j.jacc.2008.01.073

