# Correction: 2023 Korean Society of Echocardiography position paper for diagnosis and management of valvular heart disease, part I: aortic valve disease

**DOI:** 10.1186/s44348-024-00039-w

**Published:** 2024-11-08

**Authors:** Sun Hwa Lee, Se Jung Yoon, Byung Joo Sun, Hyue Mee Kim, Hyung Yoon Kim, Sahmin Lee, Chi Young Shim, Eun Kyoung Kim, Dong Hyuk Cho, Jun Bean Park, Jeong Sook Seo, Jung Woo Son, In Cheol Kim, Sang Hyun Lee, Ran Heo, Hyun Jung Lee, Jae Hyeong Park, Jong Min Song, Sang Chol Lee, Hyungseop Kim, Duk Hyun Kang, Jong Won Ha, Kye Hun Kim, on the behalf of the Korean Valve Survey Investigators

**Affiliations:** 1 Division of Cardiology, Department of Internal Medicine, Jeonbuk National University Medical School, Jeonbuk National University Hospital, Jeonju, Republic of Korea; 2 Division of Cardiology, National Health Insurance Service Ilsan Hospital, Goyang, Republic of Korea; 3 Division of Cardiology, Asan Medical Center, University of Ulsan College of Medicine, Seoul, Republic of Korea; 4 Division of Cardiology, Department of Internal Medicine, ChungAng University Hospital, Chung-Ang University College of Medicine, Seoul, Republic of Korea; 5 Department of Cardiovascular Medicine, Chonnam National University Hospital, Chonnam National University Medical School, Gwangju, Republic of Korea; 6 Division of Cardiology, Severance Cardiovascular Hospital, Yonsei University College of Medicine, Seoul, Republic of Korea; 7 Division of Cardiology, Department of Medicine, Samsung Medical Center, Sungkyunkwan University School of Medicine, Seoul, Republic of Korea; 8 Heart Vascular Stroke Institute, Samsung Medical Center, Seoul, Republic of Korea; 9 Division of Cardiology, Department of Internal Medicine, Korea University Anam Hospital, Seoul, Republic of Korea; 10 Division of Cardiology, Department of Internal Medicine, Seoul National University Hospital, Seoul, Republic of Korea; 11 Division of Cardiology, Department of Internal Medicine, Inje University Busan Paik Hospital, Inje University College of Medicine, Busan, Republic of Korea; 12 Division of Cardiology, Department of Internal Medicine, Yonsei University Wonju College of Medicine, Wonju, Republic of Korea; 13 Department of Internal Medicine, Keimyung University Dongsan Medical Center, Daegu, Republic of Korea; 14 Division of Cardiology, Pusan National Yangsan Hospital, Pusan National University School of Medi Cine, Busan, Republic of Korea; 15 Research Institute for Convergence of Bio Medical Science and Technology, Pusan National Yangsan Hospital, Busan, Republic of Korea; 16 Division of Cardiology, Department of Internal Medicine, Hanyang University College of Medicine, Seoul, Republic of Korea; 17 Division of Cardiology, Department of Internal Medicine, Chungnam National University Hospital, Chungnam National University College of Medicine, Daejeon, Republic of Korea; 18 Regional Cardiocerebrovascular Center, Chungnam National University Hospital, Daejeon, Republic of Korea

**Correction** **:** **J Cardiovasc Imaging 32, 11 (2024)**

**https://doi.org/10.1186/s44348-024-00019-0**

During the publication process of the article [1], the affiliation of the author Kye Hun Kim was not shown correctly:

The incorrect affiliation:

Department of Cardiovascular Medicine, Chonnam National University Hospital, Gwangju, Republic of Korea

The Correction affiliation:

Department of Cardiovascular Medicine, Chonnam National University Hospital, Chonnam National University Medical School, Gwangju, Republic of Korea

The original article has been updated.
